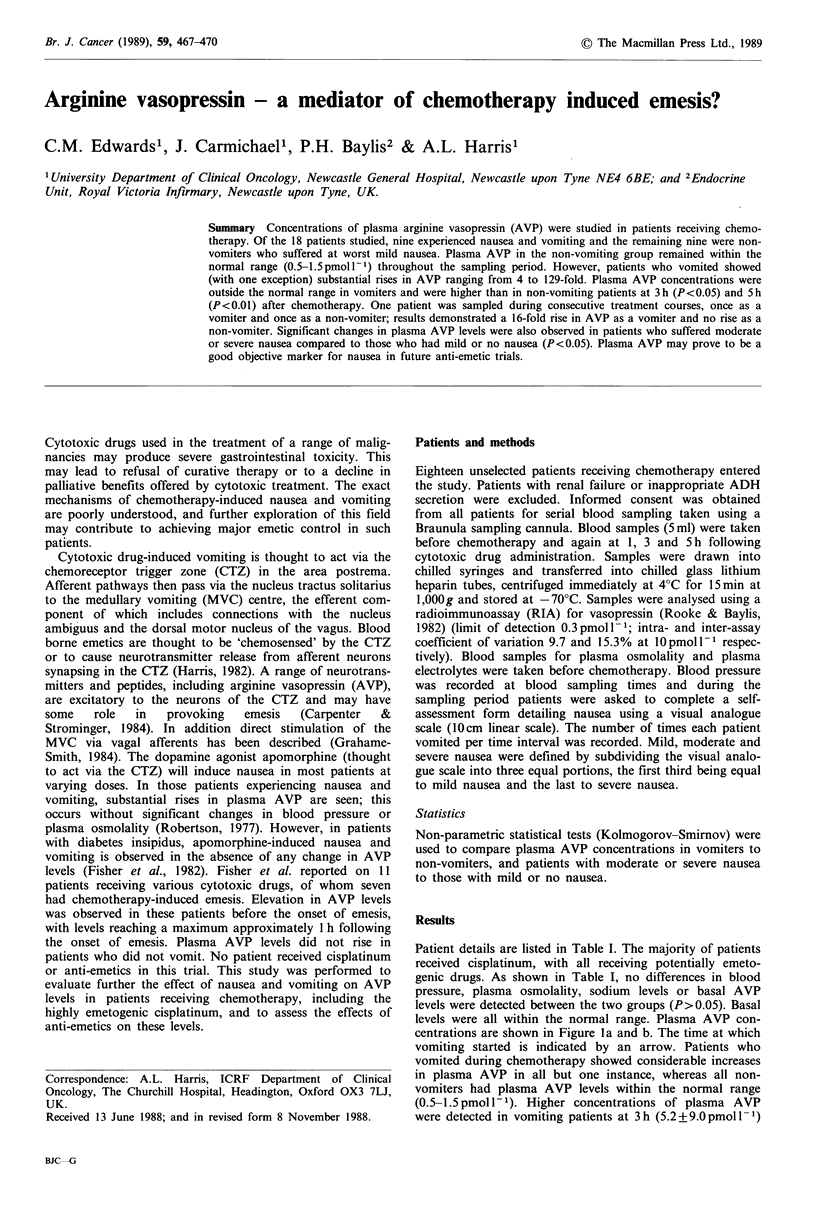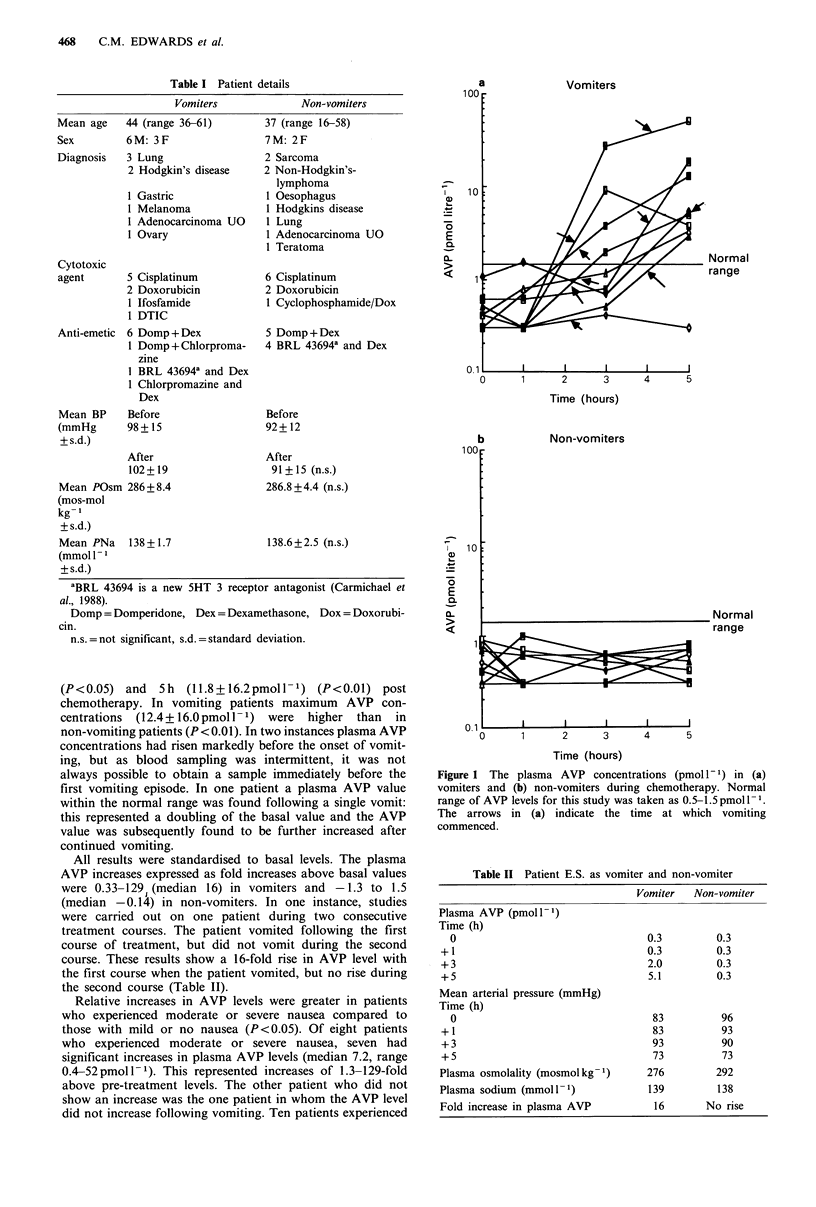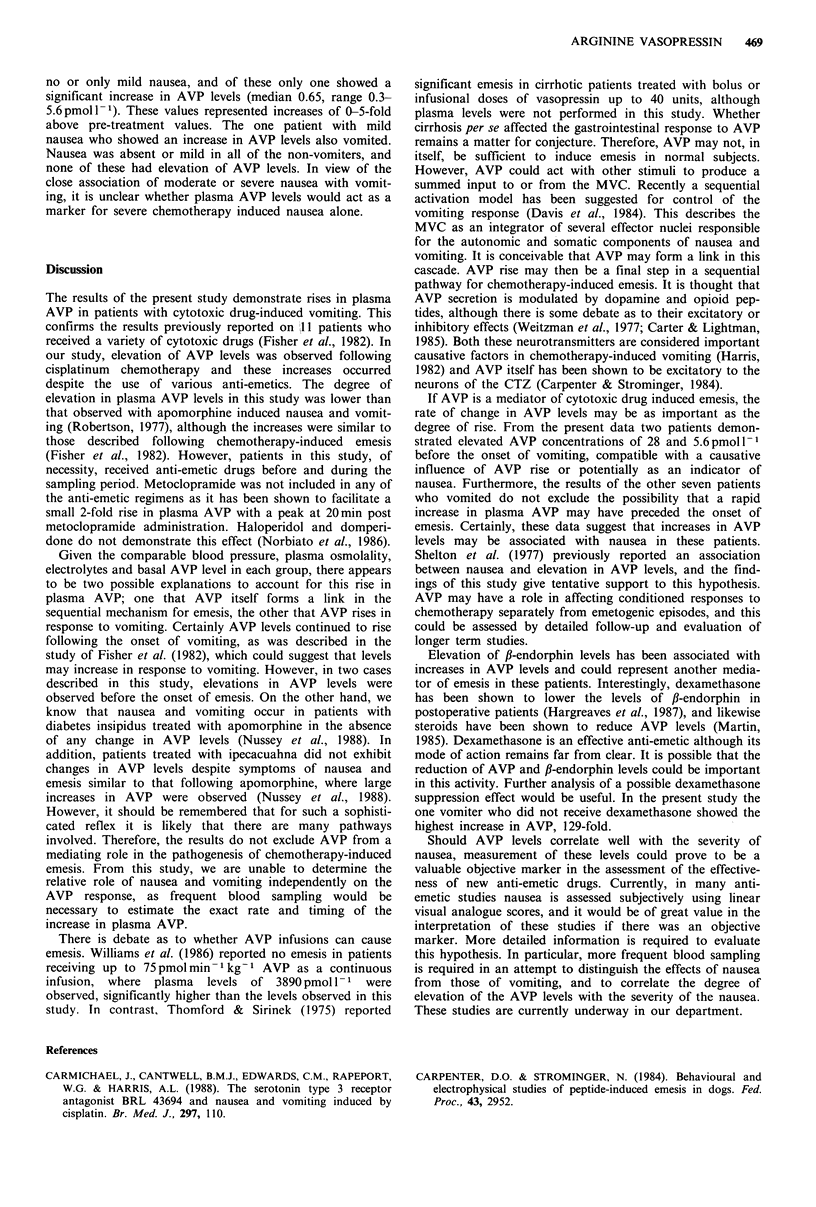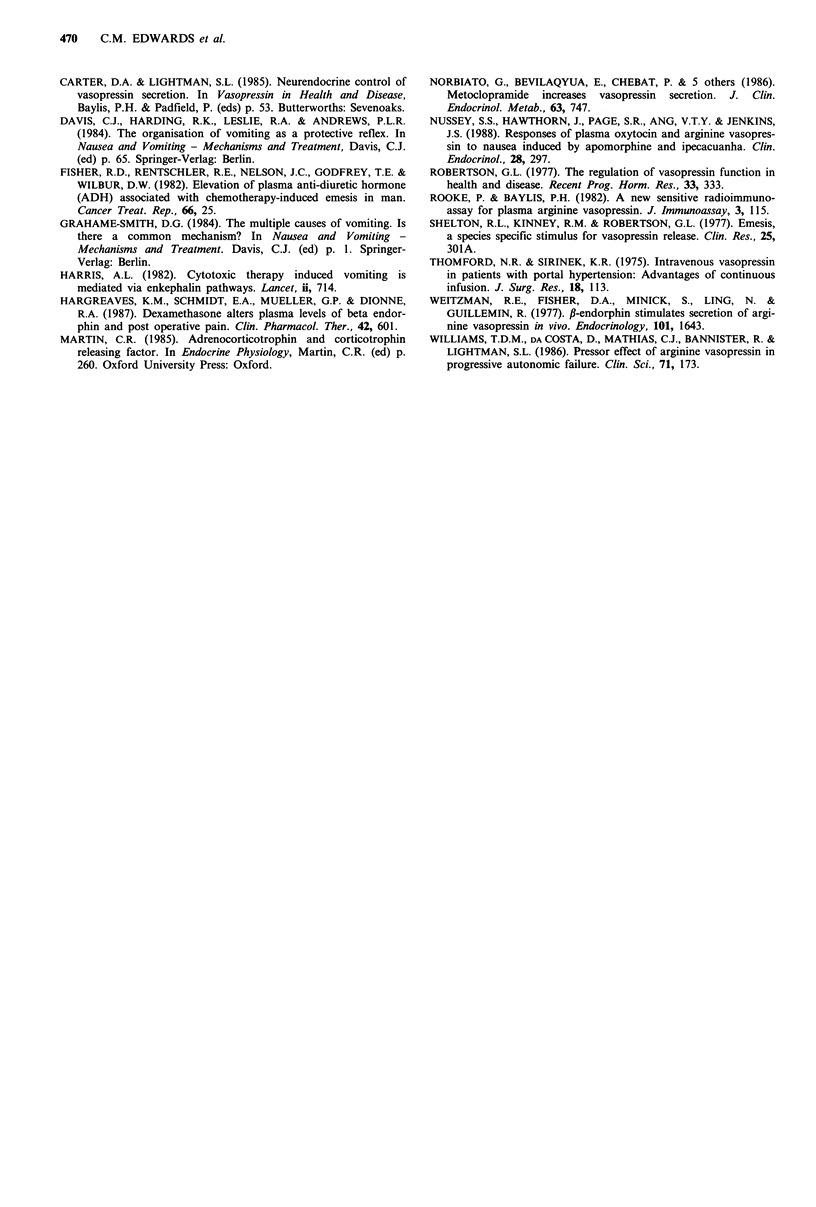# Arginine vasopressin--a mediator of chemotherapy induced emesis?

**DOI:** 10.1038/bjc.1989.96

**Published:** 1989-03

**Authors:** C. M. Edwards, J. Carmichael, P. H. Baylis, A. L. Harris

**Affiliations:** University Department of Clinical Oncology, Newcastle General Hospital, Newcastle upon Tyne, UK.

## Abstract

Concentrations of plasma arginine vasopressin (AVP) were studied in patients receiving chemotherapy. Of the 18 patients studied, nine experienced nausea and vomiting and the remaining nine were nonvomiters who suffered at worst mild nausea. Plasma AVP in the non-vomiting group remained within the normal range (0.5-1.5 pmol 1(-1] throughout the sampling period. However, patients who vomited showed (with one exception) substantial rises in AVP ranging from 4 to 129-fold. Plasma AVP concentrations were outside the normal range in vomiters and were higher than in non-vomiting patients at 3 h (P less than 0.05) and 5h (P less than 0.01) after chemotherapy. One patient was sampled during consecutive treatment courses, once as a vomiter and once as a non-vomiter; results demonstrated a 16-fold rise in AVP as a vomiter and no rise as a non-vomiter. Significant changes in plasma AVP levels were also observed in patients who suffered moderate or severe nausea compared to those who had mild or no nausea (P less than 0.05). Plasma AVP may prove to be a good objective marker for nausea in future anti-emetic trials.


Br. J. Cancer (1989), 59, 467–470 © The Macmillan Press Ltd., 1989

### Arginine vasopressin – a mediator of chemotherapy induced emesis?

C.M. Edwards[1], J. Carmichael[1], P.H. Baylis[2] & A.L. Harris[1]

[1]*University Department of Clinical Oncology, Newcastle General Hospital, Newcastle upon Tyne NE4 6BE; and* [2]*Endocrine Unit, Royal Victoria Infirmary, Newcastle upon Tyne, UK.*

**Summary** Concentrations of plasma arginine vasopressin (AVP) were studied in patients receiving chemotherapy. Of the 18 patients studied, nine experienced nausea and vomiting and the remaining nine were non-vomiters who suffered at worst mild nausea. Plasma AVP in the non-vomiting group remained within the normal range (0.5–1.5 pmol l$^{-1}$) throughout the sampling period. However, patients who vomited showed (with one exception) substantial rises in AVP ranging from 4 to 129-fold. Plasma AVP concentrations were outside the normal range in vomiters and were higher than in non-vomiting patients at 3 h ($P<0.05$) and 5 h ($P<0.01$) after chemotherapy. One patient was sampled during consecutive treatment courses, once as a vomiter and once as a non-vomiter; results demonstrated a 16-fold rise in AVP as a vomiter and no rise as a non-vomiter. Significant changes in plasma AVP levels were also observed in patients who suffered moderate or severe nausea compared to those who had mild or no nausea ($P<0.05$). Plasma AVP may prove to be a good objective marker for nausea in future anti-emetic trials.

Cytotoxic drugs used in the treatment of a range of malignancies may produce severe gastrointestinal toxicity. This may lead to refusal of curative therapy or to a decline in palliative benefits offered by cytotoxic treatment. The exact mechanisms of chemotherapy-induced nausea and vomiting are poorly understood, and further exploration of this field may contribute to achieving major emetic control in such patients.

Cytotoxic drug-induced vomiting is thought to act via the chemoreceptor trigger zone (CTZ) in the area postrema. Afferent pathways then pass via the nucleus tractus solitarius to the medullary vomiting (MVC) centre, the efferent component of which includes connections with the nucleus ambiguus and the dorsal motor nucleus of the vagus. Blood borne emetics are thought to be 'chemosensed' by the CTZ or to cause neurotransmitter release from afferent neurons synapsing in the CTZ (Harris, 1982). A range of neurotransmitters and peptides, including arginine vasopressin (AVP), are excitatory to the neurons of the CTZ and may have some role in provoking emesis (Carpenter & Strominger, 1984). In addition direct stimulation of the MVC via vagal afferents has been described (Grahame-Smith, 1984). The dopamine agonist apomorphine (thought to act via the CTZ) will induce nausea in most patients at varying doses. In those patients experiencing nausea and vomiting, substantial rises in plasma AVP are seen; this occurs without significant changes in blood pressure or plasma osmolality (Robertson, 1977). However, in patients with diabetes insipidus, apomorphine-induced nausea and vomiting is observed in the absence of any change in AVP levels (Fisher *et al.*, 1982). Fisher *et al.* reported on 11 patients receiving various cytotoxic drugs, of whom seven had chemotherapy-induced emesis. Elevation in AVP levels was observed in these patients before the onset of emesis, with levels reaching a maximum approximately 1 h following the onset of emesis. Plasma AVP levels did not rise in patients who did not vomit. No patient received cisplatinum or anti-emetics in this trial. This study was performed to evaluate further the effect of nausea and vomiting on AVP levels in patients receiving chemotherapy, including the highly emetogenic cisplatinum, and to assess the effects of anti-emetics on these levels.

Correspondence: A.L. Harris, ICRF Department of Clinical Oncology, The Churchill Hospital, Headington, Oxford OX3 7LJ, UK.
Received 13 June 1988; and in revised form 8 November 1988.

### Patients and methods

Eighteen unselected patients receiving chemotherapy entered the study. Patients with renal failure or inappropriate ADH secretion were excluded. Informed consent was obtained from all patients for serial blood sampling taken using a Braunula sampling cannula. Blood samples (5 ml) were taken before chemotherapy and again at 1, 3 and 5 h following cytotoxic drug administration. Samples were drawn into chilled syringes and transferred into chilled glass lithium heparin tubes, centrifuged immediately at 4°C for 15 min at 1,000 *g* and stored at −70°C. Samples were analysed using a radioimmunoassay (RIA) for vasopressin (Rooke & Baylis, 1982) (limit of detection 0.3 pmol l$^{-1}$; intra- and inter-assay coefficient of variation 9.7 and 15.3% at 10 pmol l$^{-1}$ respectively). Blood samples for plasma osmolality and plasma electrolytes were taken before chemotherapy. Blood pressure was recorded at blood sampling times and during the sampling period patients were asked to complete a self-assessment form detailing nausea using a visual analogue scale (10 cm linear scale). The number of times each patient vomited per time interval was recorded. Mild, moderate and severe nausea were defined by subdividing the visual analogue scale into three equal portions, the first third being equal to mild nausea and the last to severe nausea.

### *Statistics*

Non-parametric statistical tests (Kolmogorov–Smirnov) were used to compare plasma AVP concentrations in vomiters to non-vomiters, and patients with moderate or severe nausea to those with mild or no nausea.

### Results

Patient details are listed in Table I. The majority of patients received cisplatinum, with all receiving potentially emetogenic drugs. As shown in Table I, no differences in blood pressure, plasma osmolality, sodium levels or basal AVP levels were detected between the two groups ($P>0.05$). Basal levels were all within the normal range. Plasma AVP concentrations are shown in Figure 1a and b. The time at which vomiting started is indicated by an arrow. Patients who vomited during chemotherapy showed considerable increases in plasma AVP in all but one instance, whereas all non-vomiters had plasma AVP levels within the normal range (0.5–1.5 pmol l$^{-1}$). Higher concentrations of plasma AVP were detected in vomiting patients at 3 h (5.2±9.0 pmol l$^{-1}$)

**Table I** Patient details

| | *Vomiters* | *Non-vomiters* |
|---|---|---|
| Mean age | 44 (range 36–61) | 37 (range 16–58) |
| Sex | 6 M: 3 F | 7 M: 2 F |
| Diagnosis | 3 Lung<br>2 Hodgkin's disease<br>1 Gastric<br>1 Melanoma<br>1 Adenocarcinoma UO<br>1 Ovary | 2 Sarcoma<br>2 Non-Hodgkin's-lymphoma<br>1 Oesophagus<br>1 Hodgkins disease<br>1 Lung<br>1 Adenocarcinoma UO<br>1 Teratoma |
| Cytotoxic agent | 5 Cisplatinum<br>2 Doxorubicin<br>1 Ifosfamide<br>1 DTIC | 6 Cisplatinum<br>2 Doxorubicin<br>1 Cyclophosphamide/Dox |
| Anti-emetic | 6 Domp + Dex<br>1 Domp + Chlorpromazine<br>1 BRL 43694[a] and Dex<br>1 Chlorpromazine and Dex | 5 Domp + Dex<br>4 BRL 43694[a] and Dex |
| Mean BP (mmHg ±s.d.) | Before<br>98±15<br>After<br>102±19 | Before<br>92±12<br>After<br>91±15 (n.s.) |
| Mean *P*Osm (mos-mol $kg^{-1}$ ±s.d.) | 286±8.4 | 286.8±4.4 (n.s.) |
| Mean *P*Na ($mmol\,l^{-1}$ ±s.d.) | 138±1.7 | 138.6±2.5 (n.s.) |

[a]BRL 43694 is a new 5HT 3 receptor antagonist (Carmichael *et al.*, 1988).
Domp = Domperidone, Dex = Dexamethasone, Dox = Doxorubicin.
n.s. = not significant, s.d. = standard deviation.

($P<0.05$) and 5h ($11.8\pm16.2\,pmol\,l^{-1}$) ($P<0.01$) post chemotherapy. In vomiting patients maximum AVP concentrations ($12.4\pm16.0\,pmol\,l^{-1}$) were higher than in non-vomiting patients ($P<0.01$). In two instances plasma AVP concentrations had risen markedly before the onset of vomiting, but as blood sampling was intermittent, it was not always possible to obtain a sample immediately before the first vomiting episode. In one patient a plasma AVP value within the normal range was found following a single vomit: this represented a doubling of the basal value and the AVP value was subsequently found to be further increased after continued vomiting.

All results were standardised to basal levels. The plasma AVP increases expressed as fold increases above basal values were 0.33–129 (median 16) in vomiters and −1.3 to 1.5 (median −0.14) in non-vomiters. In one instance, studies were carried out on one patient during two consecutive treatment courses. The patient vomited following the first course of treatment, but did not vomit during the second course. These results show a 16-fold rise in AVP level with the first course when the patient vomited, but no rise during the second course (Table II).

Relative increases in AVP levels were greater in patients who experienced moderate or severe nausea compared to those with mild or no nausea ($P<0.05$). Of eight patients who experienced moderate or severe nausea, seven had significant increases in plasma AVP levels (median 7.2, range 0.4–52 $pmol\,l^{-1}$). This represented increases of 1.3–129-fold above pre-treatment levels. The other patient who did not show an increase was the one patient in whom the AVP level did not increase following vomiting. Ten patients experienced

**Figure 1** The plasma AVP concentrations ($pmol\,l^{-1}$) in (**a**) vomiters and (**b**) non-vomiters during chemotherapy. Normal range of AVP levels for this study was taken as 0.5–1.5 $pmol\,l^{-1}$. The arrows in (**a**) indicate the time at which vomiting commenced.

**Table II** Patient E.S. as vomiter and non-vomiter

| | *Vomiter* | *Non-vomiter* |
|---|---|---|
| Plasma AVP ($pmol\,l^{-1}$) | | |
| Time (h) | | |
| 0 | 0.3 | 0.3 |
| +1 | 0.3 | 0.3 |
| +3 | 2.0 | 0.3 |
| +5 | 5.1 | 0.3 |
| Mean arterial pressure (mmHg) | | |
| Time (h) | | |
| 0 | 83 | 96 |
| +1 | 83 | 93 |
| +3 | 93 | 90 |
| +5 | 73 | 73 |
| Plasma osmolality ($mosmol\,kg^{-1}$) | 276 | 292 |
| Plasma sodium ($mmol\,l^{-1}$) | 139 | 138 |
| Fold increase in plasma AVP | 16 | No rise |

no or only mild nausea, and of these only one showed a significant increase in AVP levels (median 0.65, range 0.3–5.6 pmol l$^{-1}$). These values represented increases of 0–5-fold above pre-treatment values. The one patient with mild nausea who showed an increase in AVP levels also vomited. Nausea was absent or mild in all of the non-vomiters, and none of these had elevation of AVP levels. In view of the close association of moderate or severe nausea with vomiting, it is unclear whether plasma AVP levels would act as a marker for severe chemotherapy induced nausea alone.

### Discussion

The results of the present study demonstrate rises in plasma AVP in patients with cytotoxic drug-induced vomiting. This confirms the results previously reported on 11 patients who received a variety of cytotoxic drugs (Fisher *et al.*, 1982). In our study, elevation of AVP levels was observed following cisplatinum chemotherapy and these increases occurred despite the use of various anti-emetics. The degree of elevation in plasma AVP levels in this study was lower than that observed with apomorphine induced nausea and vomiting (Robertson, 1977), although the increases were similar to those described following chemotherapy-induced emesis (Fisher *et al.*, 1982). However, patients in this study, of necessity, received anti-emetic drugs before and during the sampling period. Metoclopramide was not included in any of the anti-emetic regimens as it has been shown to facilitate a small 2-fold rise in plasma AVP with a peak at 20 min post metoclopramide administration. Haloperidol and domperidone do not demonstrate this effect (Norbiato *et al.*, 1986).

Given the comparable blood pressure, plasma osmolality, electrolytes and basal AVP level in each group, there appears to be two possible explanations to account for this rise in plasma AVP; one that AVP itself forms a link in the sequential mechanism for emesis, the other that AVP rises in response to vomiting. Certainly AVP levels continued to rise following the onset of vomiting, as was described in the study of Fisher *et al.* (1982), which could suggest that levels may increase in response to vomiting. However, in two cases described in this study, elevations in AVP levels were observed before the onset of emesis. On the other hand, we know that nausea and vomiting occur in patients with diabetes insipidus treated with apomorphine in the absence of any change in AVP levels (Nussey *et al.*, 1988). In addition, patients treated with ipecacuahna did not exhibit changes in AVP levels despite symptoms of nausea and emesis similar to that following apomorphine, where large increases in AVP were observed (Nussey *et al.*, 1988). However, it should be remembered that for such a sophisticated reflex it is likely that there are many pathways involved. Therefore, the results do not exclude AVP from a mediating role in the pathogenesis of chemotherapy-induced emesis. From this study, we are unable to determine the relative role of nausea and vomiting independently on the AVP response, as frequent blood sampling would be necessary to estimate the exact rate and timing of the increase in plasma AVP.

There is debate as to whether AVP infusions can cause emesis. Williams *et al.* (1986) reported no emesis in patients receiving up to 75 pmol min$^{-1}$ kg$^{-1}$ AVP as a continuous infusion, where plasma levels of 3890 pmol l$^{-1}$ were observed, significantly higher than the levels observed in this study. In contrast, Thomford & Sirinek (1975) reported significant emesis in cirrhotic patients treated with bolus or infusional doses of vasopressin up to 40 units, although plasma levels were not performed in this study. Whether cirrhosis *per se* affected the gastrointestinal response to AVP remains a matter for conjecture. Therefore, AVP may not, in itself, be sufficient to induce emesis in normal subjects. However, AVP could act with other stimuli to produce a summed input to or from the MVC. Recently a sequential activation model has been suggested for control of the vomiting response (Davis *et al.*, 1984). This describes the MVC as an integrator of several effector nuclei responsible for the autonomic and somatic components of nausea and vomiting. It is conceivable that AVP may form a link in this cascade. AVP rise may then be a final step in a sequential pathway for chemotherapy-induced emesis. It is thought that AVP secretion is modulated by dopamine and opioid peptides, although there is some debate as to their excitatory or inhibitory effects (Weitzman *et al.*, 1977; Carter & Lightman, 1985). Both these neurotransmitters are considered important causative factors in chemotherapy-induced vomiting (Harris, 1982) and AVP itself has been shown to be excitatory to the neurons of the CTZ (Carpenter & Strominger, 1984).

If AVP is a mediator of cytotoxic drug induced emesis, the rate of change in AVP levels may be as important as the degree of rise. From the present data two patients demonstrated elevated AVP concentrations of 28 and 5.6 pmol l$^{-1}$ before the onset of vomiting, compatible with a causative influence of AVP rise or potentially as an indicator of nausea. Furthermore, the results of the other seven patients who vomited do not exclude the possibility that a rapid increase in plasma AVP may have preceded the onset of emesis. Certainly, these data suggest that increases in AVP levels may be associated with nausea in these patients. Shelton *et al.* (1977) previously reported an association between nausea and elevation in AVP levels, and the findings of this study give tentative support to this hypothesis. AVP may have a role in affecting conditioned responses to chemotherapy separately from emetogenic episodes, and this could be assessed by detailed follow-up and evaluation of longer term studies.

Elevation of $\beta$-endorphin levels has been associated with increases in AVP levels and could represent another mediator of emesis in these patients. Interestingly, dexamethasone has been shown to lower the levels of $\beta$-endorphin in postoperative patients (Hargreaves *et al.*, 1987), and likewise steroids have been shown to reduce AVP levels (Martin, 1985). Dexamethasone is an effective anti-emetic although its mode of action remains far from clear. It is possible that the reduction of AVP and $\beta$-endorphin levels could be important in this activity. Further analysis of a possible dexamethasone suppression effect would be useful. In the present study the one vomiter who did not receive dexamethasone showed the highest increase in AVP, 129-fold.

Should AVP levels correlate well with the severity of nausea, measurement of these levels could prove to be a valuable objective marker in the assessment of the effectiveness of new anti-emetic drugs. Currently, in many anti-emetic studies nausea is assessed subjectively using linear visual analogue scores, and it would be of great value in the interpretation of these studies if there was an objective marker. More detailed information is required to evaluate this hypothesis. In particular, more frequent blood sampling is required in an attempt to distinguish the effects of nausea from those of vomiting, and to correlate the degree of elevation of the AVP levels with the severity of the nausea. These studies are currently underway in our department.

### References

CARMICHAEL, J., CANTWELL, B.M.J., EDWARDS, C.M., RAPEPORT, W.G. & HARRIS, A.L. (1988). The serotonin type 3 receptor antagonist BRL 43694 and nausea and vomiting induced by cisplatin. *Br. Med. J.*, **297**, 110.

CARPENTER, D.O. & STROMINGER, N. (1984). Behavioural and electrophysical studies of peptide-induced emesis in dogs. *Fed. Proc.*, **43**, 2952.

CARTER, D.A. & LIGHTMAN, S.L. (1985). Neurendocrine control of vasopressin secretion. In *Vasopressin in Health and Disease*, Baylis, P.H. & Padfield, P. (eds) p. 53. Butterworths: Sevenoaks.

DAVIS, C.J., HARDING, R.K., LESLIE, R.A. & ANDREWS, P.L.R. (1984). The organisation of vomiting as a protective reflex. In *Nausea and Vomiting – Mechanisms and Treatment*, Davis, C.J. (ed) p. 65. Springer-Verlag: Berlin.

FISHER, R.D., RENTSCHLER, R.E., NELSON, J.C., GODFREY, T.E. & WILBUR, D.W. (1982). Elevation of plasma anti-diuretic hormone (ADH) associated with chemotherapy-induced emesis in man. *Cancer Treat. Rep.*, **66**, 25.

GRAHAME-SMITH, D.G. (1984). The multiple causes of vomiting. Is there a common mechanism? In *Nausea and Vomiting – Mechanisms and Treatment*. Davis, C.J. (ed) p. 1. Springer-Verlag: Berlin.

HARRIS, A.L. (1982). Cytotoxic therapy induced vomiting is mediated via enkephalin pathways. *Lancet*, **ii**, 714.

HARGREAVES, K.M., SCHMIDT, E.A., MUELLER, G.P. & DIONNE, R.A. (1987). Dexamethasone alters plasma levels of beta endorphin and post operative pain. *Clin. Pharmacol. Ther.*, **42**, 601.

MARTIN, C.R. (1985). Adrenocorticotrophin and corticotrophin releasing factor. In *Endocrine Physiology*, Martin, C.R. (ed) p. 260. Oxford University Press: Oxford.

NORBIATO, G., BEVILAQYUA, E., CHEBAT, P. & 5 others (1986). Metoclopramide increases vasopressin secretion. *J. Clin. Endocrinol. Metab.*, **63**, 747.

NUSSEY, S.S., HAWTHORN, J., PAGE, S.R., ANG, V.T.Y. & JENKINS, J.S. (1988). Responses of plasma oxytocin and arginine vasopressin to nausea induced by apomorphine and ipecacuanha. *Clin. Endocrinol.*, **28**, 297.

ROBERTSON, G.L. (1977). The regulation of vasopressin function in health and disease. *Recent Prog. Horm. Res.*, **33**, 333.

ROOKE, P. & BAYLIS, P.H. (1982). A new sensitive radioimmunoassay for plasma arginine vasopressin. *J. Immunoassay*, **3**, 115.

SHELTON, R.L., KINNEY, R.M. & ROBERTSON, G.L. (1977). Emesis, a species specific stimulus for vasopressin release. *Clin. Res.*, **25**, 301A.

THOMFORD, N.R. & SIRINEK, K.R. (1975). Intravenous vasopressin in patients with portal hypertension: Advantages of continuous infusion. *J. Surg. Res.*, **18**, 113.

WEITZMAN, R.E., FISHER, D.A., MINICK, S., LING, N. & GUILLEMIN, R. (1977). β-endorphin stimulates secretion of arginine vasopressin *in vivo*. *Endocrinology*, **101**, 1643.

WILLIAMS, T.D.M., DA COSTA, D., MATHIAS, C.J., BANNISTER, R. & LIGHTMAN, S.L. (1986). Pressor effect of arginine vasopressin in progressive autonomic failure. *Clin. Sci.*, **71**, 173.